# Supplementary material for: CD6 regulates CD4 T follicular helper cell differentiation and humoral immunity during murine coronavirus infection
Source: J Virol. 2024 Dec 16;99(1):e01864-24. doi: 10.1128/jvi.01864-24 (PMC11784103; doi:10.1128/jvi.01864-24)
Supplement: Supplemental figures — Figure S1 to S3. [file jvi.01864-24-s0001.docx]

**Supplemental Figure 1: Representative gating strategy of mCoV infected cLNs at day 7 PI. S**inglet cells that fell into a lymphocyte FSC, SSC gate were analyzed for cell viability using a live/dead dye. CD45+ live cells were then first A) analyzed for CD3e expression followed by CD4 or CD8 expression. CD4 expressing T cells were then analyzed for activation CD44 and T_FH_ cells by CXCR5 and PD1 co-expression. B) CD45+ cells were also analyzed for CD19, followed by GL7 expression or IgD expression. Finally, IgD- B cells were examined for CD138.

**Supplemental Figure 2: CD6 is expressed on CD4 and CD8 T cells.** A) cLNs and B) brains from naïve WT (n=4) and CD6 KO (n=2) mice were analyzed by flow cytometry for CD6 expression on the indicated population. Representative histograms of CD6 expression in the naïve cLNs are shown in the left panel of (A). C) Representative flow cytometry plots from the naïve cLNs (left) and brain (right) demonstrating no detectable CD6 expression on CD45^-^ cells. D) Representative histograms of cLNs were taken at day 4 (left) and 7 (right) PI of CD6 expression on live cells with T cells gated out. E) CD6 expression on activated T cells at day 4PI in the cLNs was confirmed by flow cytometry. All cells were identified by flow cytometry using the gating scheme depicted in Supplemental Figure 1. Each data point represents an individual mouse and representative histograms and flow plots are from an individual WT (red) and CD6 KO (grey) mouse.

**Supplemental Figure 3: CD6 KO mice have more T cells in the liver after mCoV infection.** Livers from IC infected WT and CD6 KO mice were analyzed by qRT-PCR for **A)** *Ifng* transcripts at day 7 PI as well as viral nucleocapsid transcripts, which were below the limit of detection. Livers were also assessed by flow cytometry and accessed for **B)** total CD4 T cells (left) and CD44 expression by CD4 T cells (right) as well as **C)** total CD8 T cells (left) and CD44+ expression on CD8 T cells (right). For all graphs each data points represents an individual mouse from two independent experiments. Significance was determined using an unpaired T test and denoted as * for p<0.05, ** for p<0.01, *** for p<0.001,and **** for p<0.0001.
